# Resetting the T Cell Compartment in Autoimmune Diseases With Autologous Hematopoietic Stem Cell Transplantation: An Update

**DOI:** 10.3389/fimmu.2018.00767

**Published:** 2018-04-20

**Authors:** Lisanne Lutter, Julia Spierings, Femke C. C. van Rhijn-Brouwer, Jacob M. van Laar, Femke van Wijk

**Affiliations:** ^1^Laboratory of Translational Immunology, Department of Pediatrics, University Medical Center Utrecht, Utrecht, Netherlands; ^2^Department of Rheumatology and Clinical Immunology, University Medical Center Utrecht, Utrecht, Netherlands; ^3^Department of Nephrology and Hypertension, University Medical Center Utrecht, Utrecht, Netherlands

**Keywords:** autologous hematopoietic stem cell transplantation, autoimmune disease, T cell reconstitution, T cell receptor repertoire, regulatory T cell

## Abstract

Autologous hematopoietic stem cell transplantation (aHSCT) for autoimmune diseases has been applied for two decades as a treatment for refractory patients with progressive disease. The rationale behind aHSCT is that high-dose immunosuppression eliminates autoreactive T and B cells, thereby resetting the immune system. Post-aHSCT the cytotoxic CD8^+^ T cells normalize *via* clonal expansion due to homeostatic proliferation within a few months. CD4^+^ T cells recover primarily *via* thymopoiesis resulting in complete renewal of the T cell receptor (TCR) repertoire which requires years or never normalize completely. The increase in naïve T cells inducing immune tolerance, renewal of especially the regulatory TCR repertoire, and a less pro-inflammatory functional profile of the CD4^+^ T cells seem essential for successful immune reconstitution inducing long-term remission. There is currently a knowledge gap regarding the immune response in tissue sites post-aHSCT, as well as disease-specific factors that may determine remission or relapse. Future studies on lymphocyte dynamics and function may pave the way for optimized conditioning regimens with a more individualized approach.

## Introduction

Autoimmune diseases in general are characterized by a loss of immune tolerance. This results in generation and activation of autoreactive T and B cells leading to inflammation and consequently tissue damage. Autologous hematopoietic stem cell transplantation (aHSCT) for autoimmune diseases aims to eliminate autoreactive T and B cells and regenerate an immune system which is self-tolerant (1). The first reported aHSCT for an autoimmune disease, systemic sclerosis (SSc), was in 1996 by Tamm et al. (2). Since then, aHSCT has also been increasingly applied for treatment-refractory patients with other progressive autoimmune diseases, including multiple sclerosis (MS), Crohn’s disease, and juvenile idiopathic arthritis (JIA). Especially for SSc and MS, the clinical efficacy has been demonstrated in phase II and III clinical trials (3–6), whereas for Crohn’s disease the clinical efficacy is much more variable (5, 7). In JIA, the need for transplantation has declined since the appearance of effective therapies (8). Though international clinical guidelines have acknowledged the value of aHSCT for selected patients with refractory disease, it generally remains a rescue therapy due to the invasive nature and risk of treatment-related mortality, as well as the continuous development of new therapeutics (9). Although aHSCT is not a curative treatment, long-term remission can be achieved, and patients with relapses are usually responsive to conventional treatment again (10).

Autologous HSCT treatment starts with mobilization of hematopoietic stem cells into the peripheral circulation using cyclophosphamide (CYC) and recombinant G-CSF. This enables apheresis of stem cells. CD34^+^ selection, to purge the collected stem cells of T cells can be performed, although it is unclear whether it has clinical benefits. The next step is the immunoablative conditioning phase. In autoimmune disease high-dose CYC and occasionally, *in vivo* purging is performed by systemic administration of antibodies such as anti-thymocyte globulin (ATG) or rituximab. Finally, the hematopoietic stem cells are reinfused, which accelerates hematopoietic reconstitution (1). Exactly how aHSCT rewires a faulty immune system is still unknown. It is unclear which cells need to be depleted and which ones are important to keep. Additionally, not all cells are depleted by aHSCT and residing cells may pose a risk of early disease relapse. Understanding the quantitative and qualitative lymphocyte dynamics in relation to clinical outcome is therefore crucial to design less toxic but efficacious targeted therapies aimed at resetting the immune balance. Here, we will discuss the latest findings on T cell reconstitution post-aHSCT for autoimmune diseases, including T cell receptor (TCR) repertoire changes, and how these findings relate to clinical efficacy.

## T Cell Reconstitution

The innate immune system recovers within weeks post-aHSCT, in contrast to the reconstitution of the adaptive immune system which can take years [for recent in-depth reviews, see Ref. (10–14)].

Generally, the peripheral lymphocyte count and subsets at baseline, before aHSCT, are similar to healthy controls. Patients with MS that clinically responded to aHSCT in a phase II clinical trial, had higher memory CD4^+^ and CD8^+^ T cell counts pre-aHSCT compared with non-responders (15) and for SSc the same trend in higher complete CD4^+^ and CD8^+^ T cell counts pre-aHSCT for the responders was observed (16). This might suggest that patients with increased peripheral CD4^+^ T cell activation pre-aHSCT may respond better to aHSCT.

### CD8^+^ T Cells

Following aHSCT, the lymphopenic environment drives lymphopenia-induced proliferation. Cytotoxic CD8^+^ T cells are the first T cells to normalize and the ratio of naïve to memory CD8^+^ T cells remains constant post-aHSCT. In patients with MS early expression (within 6 months) of the inhibitory molecule programmed cell death-1 protein (PD-1) on CD8^+^ T cells correlated with a good clinical response post-aHSCT (17). Early PD-1 expression is likely protective by maintaining peripheral immune tolerance (18).

### CD4^+^ T Cells

CD4^+^ T cell reconstitution is more dependent on thymopoiesis, and CD4^+^ T cell numbers often requires years to normalize. As a consequence, there is an inversed CD4/CD8 T cell ratio. Furthermore, following aHSCT the residual naive T cells disappear, seemingly due to rapid maturation to effector memory T cells, resulting in decreased naive and increased effector memory T cells in the first 3 months post-aHSCT (17). Naive CD4^+^ T cells increase upon thymic reactivation after several months, which results in a relative decrease of central memory CD4^+^ T cells. The CD4^+^ T cell compartment also reshapes post-aHSCT compared with baseline. Unfortunately, correlations with clinical outcomes were ambiguous. In a single arm study of 11 SSc patients receiving aHSCT, naive and memory CD4^+^ T cells remained decreased during the follow-up period of 3 years (19). All patients had a good response to treatment. Decreased CD4^+^ T cells after 9 months in both responders and non-responders was reported in another study in SSc patients (20). Faster increase of CD4^+^ T cells in non-responders was seen in two studies in SSc patients (16, 20). Furthermore, while T helper (Th) 1 and 2 cells remain unaltered in frequency, Th17 cells diminish below baseline post-aHSCT, but normalize after 6 months. Functionally, post-aHSCT the Th1 and Th17 cells show a reduced interferon-γ and interleukin (IL)-17 response, respectively (12, 15, 17, 21–25). Above mentioned changes are also observed on transcriptional level, with the transcriptional program of CD8^+^ T cells normalizing within 2 years post-aHSCT, whereas the transcriptional program of CD4^+^ T cells significantly changes post-aHSCT but does not normalize (26).

### Regulatory T Cells

Data regarding regulatory T cells is contradicting, with most studies observing an increase of regulatory T cells following transplantation, usually temporarily, although in some studies no changes or decreased relative frequencies are found (12, 15, 17, 21–25, 27, 28). Regulatory T cells of clinically responding SSc patients had increased levels of the immune regulators cytotoxic T lymphocyte-associated protein 4 (CTLA-4), a negative regulator of T cell function, and glucocorticoid-induced TNFR-related protein compared with non-responders (28). Upregulation of CTLA-4 on regulatory T cells post-aHSCT in both MS and type 1 diabetes mellitus (DM type 1) patients, and increased regulatory T cell functional marker expression such as IL-10 and transforming growth factor-β in DM type 1 patients post-aHSCT has also been observed, but in these cases without positive association with the clinical outcome (17, 24). In mice, graft-derived regulatory T cells were shown to have superior suppressive function compared with regulatory T cells that survived conditioning (29). In conclusion, renewal of the regulatory T cell compartment seems essential for long-term restoration of immune homeostasis, with qualitative changes of regulatory T cells having a more profound impact than quantitative changes, although this may differ per autoimmune disease.

## TCR Repertoire Post-Transplantation

Initially, lymphopenia-induced proliferation following transplantation results in clonal expansion of residual T cells. Restoration of a fully competent T cell compartment with a diverse TCR repertoire is depending on thymic output that starts 3–6 months following HSCT. In SSc, the total TCR repertoire diversifies post-aHSCT, irrespective of the clinical outcome (16), although with a trend to a more diverse repertoire in patients without a relapse (20). However, a recent study in SSc patients did show increased TCR diversity at 1 year post-aHSCT in responders compared with non-responders. Furthermore, low overlap in TCRβ (CDR3) clonotype before and after transplantation was observed in responders (25% overlap) but not in non-responders (60% overlap) (28). This suggests that renewal of the TCR repertoire is important for the re-establishment of immune homeostasis. The difference in TCR repertoire outcome might be explained by use of ATG in the latter study, whereas this was not used or optional in addition to CYC in the first two studies (16, 20, 28).

In MS patients renewal of the CD4^+^ T cell repertoire was observed, with the dominant CD4^+^ T cell clones present at baseline not detectable following transplantation. In contrast, dominant CD8^+^ T cell clones detectable pre-aHSCT remained present post-aHSCT, and were clonally expanded (21, 22). Especially early on, non-responding MS patients had a less diverse TCR repertoire than patients without relapse, though this difference had disappeared at 1 year post-transplantation (22). In JIA and juvenile dermatomyositis patients, the CD4^+^ T cell and FOXP3 regulatory TCR repertoire were studied separately. Compared with baseline, responders had a far more diverse regulatory T cell repertoire post-aHSCT. In contrast, patients that experienced a relapse showed an even more oligoclonal repertoire compared with baseline. The CD4^+^ TCR repertoire also diversified in responders, but less pronounced than in the regulatory T cell compartment. Similarly to MS patients, no overlapping CD4^+^ TCRs were found pre- and post-aHSCT (29).

In conclusion, even though these studies were performed in patients with different autoimmune diseases there is a consistent pattern. The TCR repertoire of CD8^+^ T cells remains relatively oligoclonal, and the same dominant TCR clones can be observed pre- and post-aHSCT without noticeable clinical consequences. This suggests these CD8^+^ T cells are not self-reactive, or are but unable to induce disease activity following transplantation. The CD4^+^ T cells are characterized by a complete renewal of the TCR repertoire, and especially an increased TCR diversity for regulatory T cells seems important for a successful induction of remission post-transplantation.

## Tissue T Cell Depletion

Almost all data regarding the immune reconstitution following aHSCT are based on cells in circulation. However, the aberrant inflammation in autoimmune diseases is primarily located in tissue sites. Recently, it was shown that in intestinal tissue of patients with refractory Crohn’s disease the TCR repertoire diversifies post-aHSCT. Approximately 20% of TCR sequences were detected pre- and 6 months to 1 year post-aHSCT, demonstrating a vast resetting of the TCR repertoire (30). The clinical impact of a local expanding TCR repertoire has not been established, but these preliminary data show a local immune response occurs following transplantation. In SSc, the TCR repertoire in affected skin is strongly oligoclonal (31). Following aHSCT, there is a reduction of skin fibrosis in responding patients (follow-up time of approximately 6 years) (16), but whether this is associated with the generation of a polyclonal TCR repertoire in the skin is unknown.

Of note, the extent of tissue penetration of ATG and other immunosuppressive agents is unclear. In mice, depletion of T cells by ATG is less efficient in peripheral lymphoid organs compared with the blood (32). It is not unlikely that tissue resident memory T cells, antigen-experienced T cells permanently residing in barrier tissues (33), are not fully depleted. Is there a need for (more) profound tissue immune cell depletion, or does maintenance of the tissue immune cells protect patients after conditioning to not succumb to infection? Future studies may shed light on the extend of tissue lymphocyte depletion following conditioning and whether this affects clinical outcome.

## The Conditioning Regimen and T Cell Reconstitution

The conditioning regimen is an important factor in immune reconstitution post-aHSCT. In a study in 13 MS patients, using CYC and ATG for conditioning, the CD4/CD8 ratio remained lower than in healthy controls, and naïve CD4^+^ T cells normalized 2 years post-aHSCT (23). In contrast, in another study in MS patients with CYC and total body irradiation employed for conditioning the CD4/CD8 ratio normalized at 2 years post-aHSCT, and an overshoot of naïve CD4^+^ T cells was observed (21). In the latter study, all patients remained in remission during the follow-up of 2–3 years, whereas in the first study 30% experienced a relapse within 3 years post-aHSCT. However, these differences might also be partially explained by differential patient selection. ATG is often implemented in the conditioning regimen (13) and can severely affect immune reconstitution. As a polyclonal antibody it targets a plethora of immune cells and induces both direct and indirect cytotoxicity. Interestingly, a recent study on ATG exposure and clinical outcome in an allogeneic HSCT setting suggests that ATG dosing should be based on lymphocyte count rather than body-weight (34). Currently, ATG dosing in aHSCT patients is not individualized and monitoring both ATG levels and lymphocytes counts could be a helpful addition to define the optimal dosing strategy.

Long-term impact of an incomplete T (and B) cell reconstitution is unknown. While reliable data regarding incomplete immunological reconstitution post-HSCT is lacking, there are indications that this is not associated with long-term morbidity. A for instance, a study assessed outcomes in rheumatoid arthritis patients that received Alemtuzumab, an anti-CD52 antibody targeting primarily T and B cells. Follow-up for 20–25 years shows an incomplete reconstitution of T and B cells, especially central memory CD4^+^ and CD8^+^ T cells and naïve B cell numbers remain reduced. The incomplete reconstitution, however, was not associated with differences in mortality, morbidity, or vaccine response when compared with rheumatoid arthritis patients that have received standard care (35). These data emphasize the need to understand the normal immune reconstitution to establish optimal conditioning regimens, as well as to compare different autoimmune diseases to identify disease-specific factors that might predict long-term remission or a relapse.

## Clinical Heterogeneity Influencing Immune Reconstitution

Unfortunately, interpretation of changes in T cell compartment after aHSCT is complicated by heterogeneity in type of autoimmune disease, conditioning regimens, patient selection, graft manipulation, post-transplantation treatment, and age- or treatment-dependent thymic involution. For instance, in some studies patients have severe immune dysregulation resulting in severe and/or progressive, treatment resistant disease. These patients have often received most available immunosuppressive therapies including biologicals before aHSCT, while in other settings (e.g., SSc) aHSCT is performed relatively early in disease. Another possible confounding factor is graft manipulation by selection of CD34^+^ stem cells. The influence of this selection on immune reconstitution is reported in several studies, and although broadly used in treatments, there is conflicting evidence of its benefits (36–38). Finally, the numbers of patients included in clinical studies generally are too small to compare outcome between responders and non-responders. Despite all confounding factors, standardized (extensive) phenotypic characterization of T cell reconstitution would be an important step forward in elucidating T cell reconstitution and relating it to treatment regimens and outcome.

## Conclusion and Future Perspectives

In the past 20 years, aHSCT has been applied for severe refractory autoimmune diseases but a comprehensive understanding of the immune reconstitution and the link with clinical outcome is still missing. Data suggest that the increase in naïve T cells, renewal of especially the regulatory TCR repertoire, and a less pro-inflammatory functional profile of the CD4^+^ T cells are essential for successful immune reconstitution and the induction of long-term remission. Future studies on lymphocyte dynamics and function may pave the way for optimized conditioning regimens with a more individualized approach.

Important outstanding questions regarding the immune reconstitution following transplantation include: (1) What is the most effective conditioning regime for each autoimmune disease? This possibly depends on which immune cells are important in the pathogenesis, and the tissue(s) where the disease manifests, thus differ per autoimmune disease. (2) Is a personalized approach to the conditioning regime, depending on the immune status pre-aHSCT, needed to improve the clinical outcome? The recent observation that in a hematologic malignancy setting ATG dosing should be based on lymphocyte count may also apply to autoimmune settings. Extensive monitoring of immune depletion/reconstitution in combination with pharmacokinetic/pharmacodynamic modeling can contribute to the development of pre- and post-transplantation precision treatment. (3) What is the local tissue effect of the conditioning regimen? It is not clear to which extent for example ATG penetrates all tissues to deplete tissue T cells, and it is unclear if and to what extent residual tissue T cells contribute to relapses of disease. Together, future studies may shed light on the fine balance between effectively destroying and renewing the immune system, but with limited toxicity.

## Author Contributions

LL, JS, FR-B, JL, and FW have discussed, written, and edited the manuscript.

## Conflict of Interest Statement

The authors declare that the research was conducted in the absence of any commercial or financial relationships that could be construed as a potential conflict of interest.

## Abbreviations

aHSCT, autologous hematopoietic stem cell transplantation; ATG, anti-thymocyte globulin; CLTA-4, cytotoxic T lymphocyte-associated protein 4; CYC, cyclophosphamide; DM type I, type I diabetes mellitus; GITR, glucocorticoid-induced TNFR-related protein; IL, interleukin; MS, multiple sclerosis; PD, pharmacodynamic; PD-1, programmed cell death-1 protein; PK, pharmacokinetic; SSc, systemic sclerosis; TCR, T cell receptor; Th, T helper; Treg, regulatory T-cell.
